# Intra‐biliary hydatid cyst rupture: A rare case report with superinfection

**DOI:** 10.1002/ccr3.8581

**Published:** 2024-03-17

**Authors:** Hanan Al‐Asbahi, Jaber H. Jaradat, Mohammad Abu‐Jeyyab, Ruba Al‐Dwairi, Bara'a W. Tailakh, Rand A. Almadadha, Ibraheem M. Alkhawaldeh, Abdulqadir J. Nashwan

**Affiliations:** ^1^ Department of Surgery Al‐Bashir Hospital Amman Jordan; ^2^ School of Medicine Mutah University Al‐Karak Jordan; ^3^ Hamad Medical Corporation Doha Qatar

**Keywords:** biliary tree, *E. Granuloses*, echinococcosis, hydatid cysts

## Abstract

**Key Clinical Message:**

Hydatid cysts, primarily found in the liver (70%), are caused by parasitic infections and can lead to severe complications such as cyst rupture. This case report describes a unique instance of a hydatid liver cyst occupying the right lobe with a communicating part with the biliary tree that ruptured showing a concurrent superinfection.

**Abstract:**

Hydatid cysts are a clinical pathology resulting from parasitic infections. They may occur in different organs of the body. However, these are mostly found in the liver (70%). This can cause significant complications including cyst rupture. Several case reports have described various hydatid cyst ruptures; however, only a few have reported an intra‐biliary hydatid cyst rupture. A 24‐year‐old male patient presented with right upper quadrant pain, jaundice, dark urine, and pale stool. Imaging studies, including Magnetic resonance cholangiopancreatography (MRCP) and computed tomography (CT), revealed a beavertail liver, cystobiliary communication and intrahepatic biliary tree‐ruptured hydatid cysts. The cyst was in the right liver lobe, which is the most common site for hydatid cysts. Surgical intervention involving laparoscopic de‐roofing and cyst removal resulted in a smooth recovery without complications. Several case reports have described various hydatid cyst ruptures; however, only a few have reported originally placed intra‐biliary hydatid cyst ruptures. This case report describes a unique instance of a hydatid liver cyst occupying the right lobe with a communicating part with the biliary tree that ruptured showing a concurrent superinfection.

## Graphical abstract

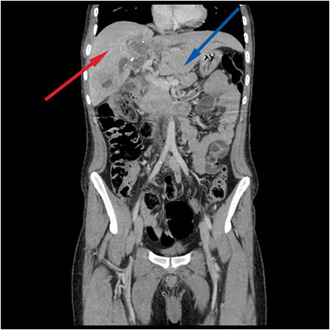

## 1 INTRODUCTION

A Hydatid cyst is an infestation that is also called echinococcosis and is caused by Echinococcus larvae. Most hydatid cysts are found in the liver in 70 percent of cases, mainly in the right lobe, which causes hydatid cysts in the hepatic duct. The World Health Organization (WHO) estimates that more than 1 million people suffer from hydatid disease. E. granulosus is endemic in India and in the Middle East, with an incidence of up to 50 per 100,000 person‐years and an estimated prevalence of 10% of the population in endemic areas.^1^, ^2^ Hydatid cysts are typically asymptomatic and last for a very long time; in most cases, they are unintentionally found during routine imaging after going unnoticed for 10–15 years. However, some patients may experience symptoms, such as nausea, vomiting, stomach pain, and jaundice. Hydatid cysts can produce symptoms when they reach 10 cm in diameter, or when complications arise, largely depending on their size and location.^3^, ^4^ Complications from hydatid disease can include secondary bacterial infection, mass effects, and ruptures. In addition, hydatid cyst ruptures fall into one of three categories: communicating ruptured cysts, containing ruptured cysts, or directly ruptured cysts.^1^

Complications associated with hydatid cysts are relatively common, occurring in 30%–60% of diagnosed and treated cases of hydatid disease. The most prevalent complication is the rupture of cysts into the biliary tree. Differential diagnoses encompass abdominal abscess, acute liver failure, biliary cirrhosis, biliary colic, and Budd‐Chiari syndrome.^5^, ^6^

In surgically treated patients, the incidence of this complication ranges from 26% to 34%. The frequency, as reported in radiology literature, varies from 3% to 17%, attributed to internal breach from high intracystic pressure or external breach due to trauma or abrupt rupture. Superinfection of hydatid cysts in the liver was observed in 24% of surgically managed cases.^6^, ^7^, ^8^

Clinical manifestations of ruptured Intrabiliary Hydatid Cyst (IBHC) may include jaundice, cholangitis, cholecystitis, liver abscess, pancreatitis, and septicemia. However, undiagnosed rupture can lead to complications such as biliary leaks, cavity infections, and obstructive jaundice.^6^

In IBHC, the right hepatic duct is frequently affected, with less impact on the left hepatic duct and confluence. Three types of hepatic hydatid cysts (HHC) are recognized, with communicating rupture being the most common, involving the seepage of hydatid cyst fluid between the endocyst and pericyst, leading to material spillage into adjacent bile ducts. Simple rupture is often asymptomatic, when progressing to obvious rupture it is associated with signs of obstruction. Direct rupture may result in the movement of cyst material into the pleural or peritoneal cavity.^9^

Abdominal ultrasound (US) and computed tomography (CT) are initial diagnostic choices for IBR. While endoscopic retrograde cholangiopancreatography (ERCP) was previously considered the gold standard, magnetic resonance cholangiopancreatography (MRCP) is gaining importance in IBR diagnosis and management. Preoperative ERCP is essential for evident IBR cases but is not recommended for large or multiple cysts, caudate or hilar lobe cysts, or minimal intrahepatic bile duct dilatation. Unlike ERCP, MRCP can detect cysto‐biliary communication and assess ducts proximal to an obstruction, preventing postoperative biliary fistula formation.^6^

Preoperative therapy involves the use of albendazole or mebendazole to reduce intracystic pressure and prevent recurrence. Endoscopic techniques, such as balloon dilatation and sphincterotomy, are employed to evacuate the bile duct. Surgical techniques include Roux‐en‐Y cysto‐jejunostomy, sutured fistula, tube drainage, and omentoplasty. Postoperative issues may include infection, sinus development, recurrence, and prolonged biliary leakage.^6^, ^10^

Several case reports have described various hydatid cyst ruptures; however, only a few have reported originally placed intra‐biliary hydatid cyst ruptures. This case report describes a unique instance of a hydatid liver cyst occupying the right lobe with a communicating part with the biliary tree that ruptured showing a concurrent superinfection. The authors have completed the CARE Checklist for this case report, attached as Data S1.

## 2 CASE PRESENTATION

A 24‐year‐old male patient presented to the emergency room with right upper quadrant pain and jaundice (yellowish discoloration of the skin and sclera) that had started 5 days prior and worsened over time. The patient also reported dark urine and pale stool.

## 3 DIAGNOSIS

**FIGURE 1:**
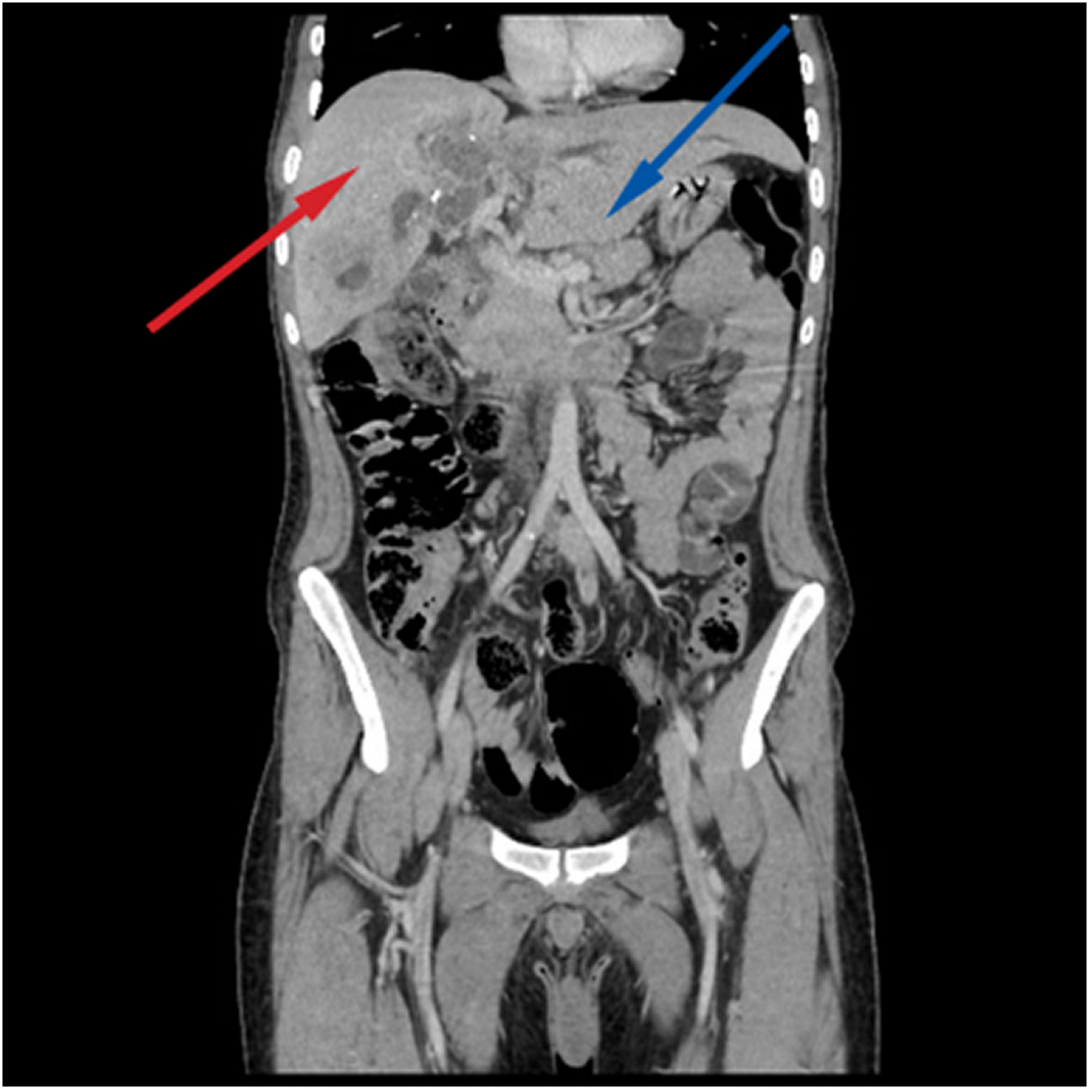
Abdominal CT (coronal) scan demonstrating elongated left lobe of the liver mimicking a beaver's tail (blue arrow) with the normal right lobe of the liver (red arrow); a beaver tail appearance liver. measuring about 15.5 cm with evidence of two occupying low‐attenuation lesions: one in segment IV and one in segment V, measuring approximately 5.5 × 4 cm and 3.5 × 5.9 cm, respectively. These lesions appeared as thick‐walled fluid‐filled lobulated structures with incomplete peripheral coarse calcifications surrounded by an ill‐defined faint heterogeneous density area and adjacent capsular retraction.

**FIGURE 2:**
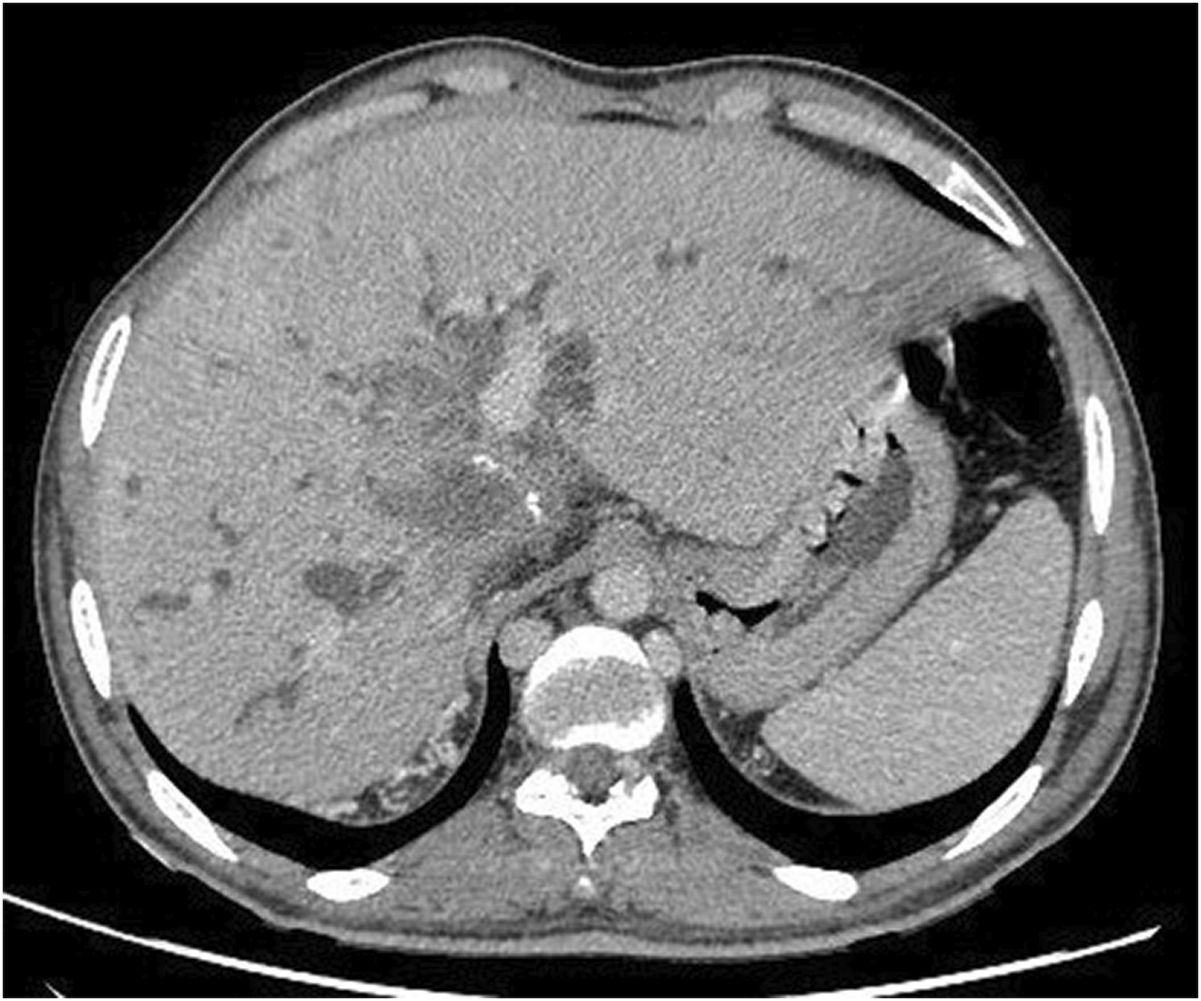
Axial abdominal CT SCAN demonstrating a low attenuating mass lesion seen in segment IV. There is a rim of fluid collection and fat stranding measuring approximately centimeters. Also, the lesion communicated with the common hepatic duct and left hepatic biliary tree, consistent with a calcified hydatid cyst.

**FIGURE 3:**
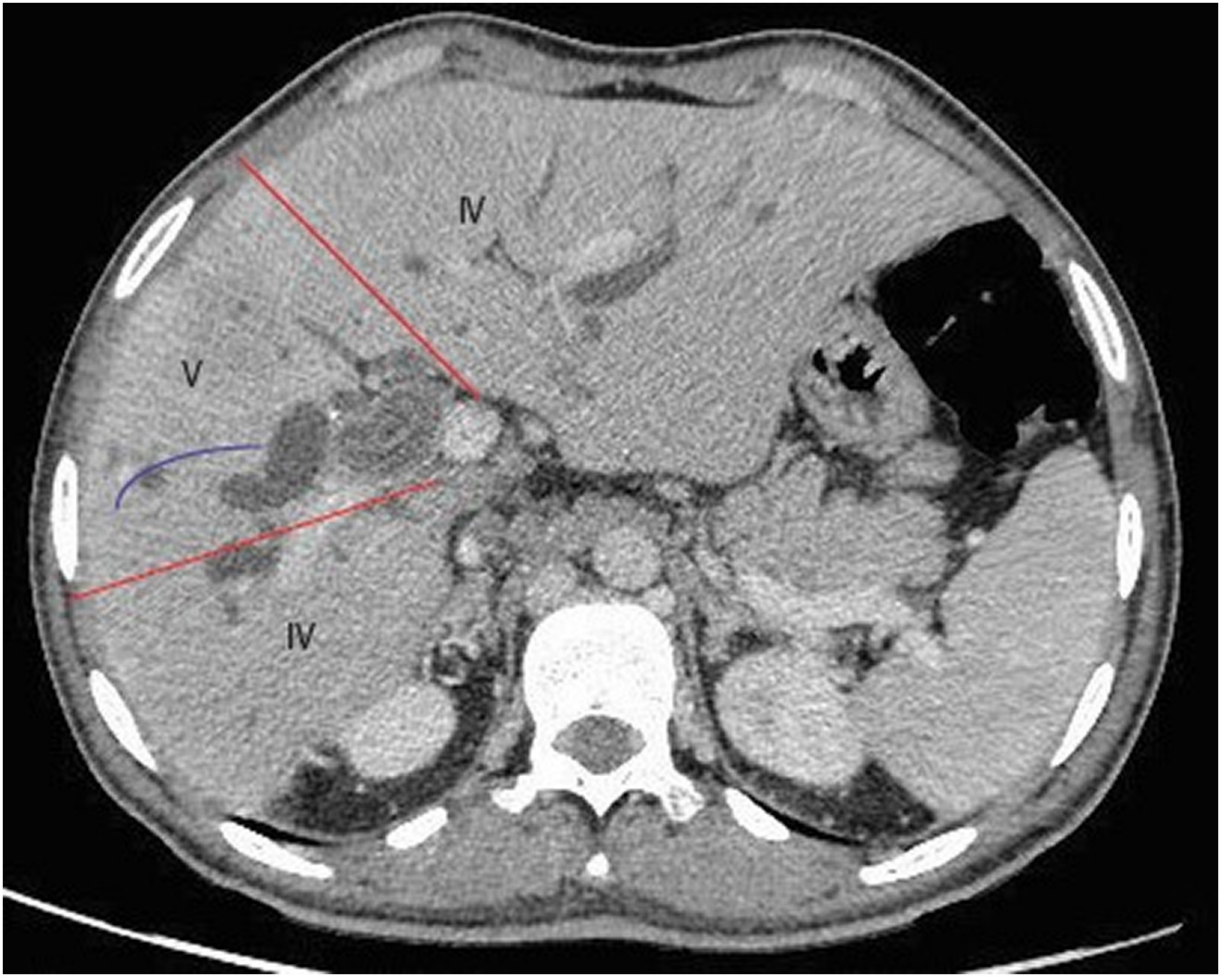
Segment 5 in abdominal axial CT SCAN showed a lobulated lesion (blue arrow). The lesion communicated with the dilated right hepatic biliary tree, and thin septations and dense material were observed within the dilated biliary tree, indicative of an intrahepatic biliary tree‐ruptured hydatid cyst.

**FIGURE 4:**
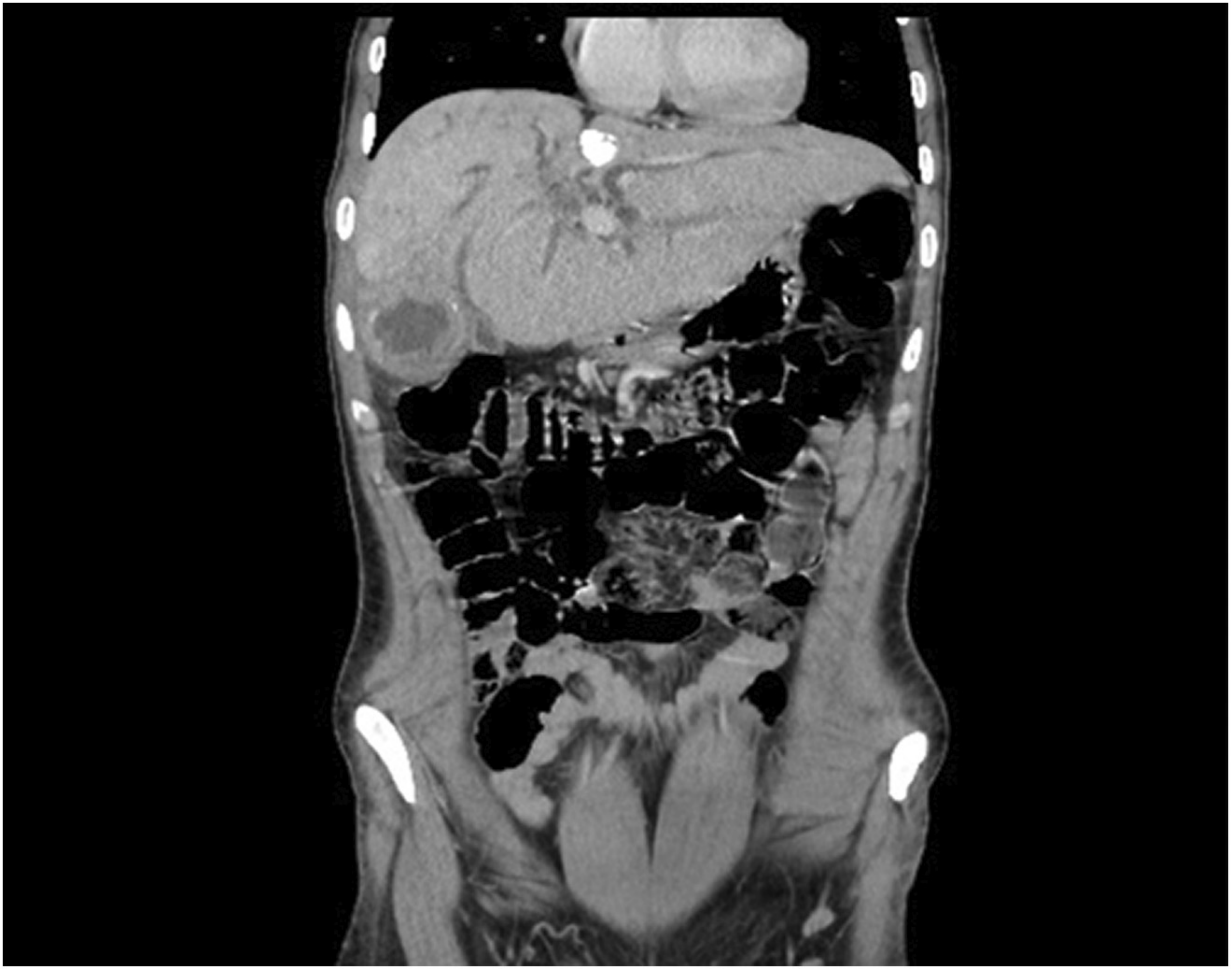
Axial abdominal CT SCAN showing a calcified lesion measuring about 2.5 × 1.3 cm in segment 4a.

Physical examination revealed stable vital signs and jaundice, but no tenderness or organomegaly. Initial US imaging showed no gall bladder stones but identified dilation of the common bile duct (CBD)to approximately 0.8 (normal range 0.4–0.6). A full blood count and basic chemistry panel indicated average Hb, WBCs, and PLT values, but high direct and total bilirubin values. On the basis of these findings, the patient was admitted to the emergency room for further investigation of obstructive jaundice. Subsequently, suboptimal MRCP and CT with intravenous contrast were performed. The findings for the MRCP and CT were beaver tail appearance liver (Figure 1) measuring about 15.5 cm with evidence of two occupying low‐attenuation lesions: one in segment IV and one in segment V, measuring approximately 5.5 × 4 cm and 3.5 × 5.9 cm, respectively. These lesions appeared as thick‐walled fluid‐filled lobulated structures with incomplete peripheral coarse calcifications surrounded by an ill‐defined faint heterogeneous density area and adjacent capsular retraction. There was also a rim of fluid collection and fat stranding measuring approximately centimeters in the axial view. The lesion in segment IV (Figure 2) communicated with the common hepatic duct and left hepatic biliary tree, consistent with a calcified hydatid cyst. The lesion in segment V (Figure 3) communicated with the dilated right hepatic biliary tree, and thin septations and dense material were observed within the dilated biliary tree, indicative of an intrahepatic biliary tree‐ruptured hydatid cyst. Multiple lymph nodes were enlarged in the porta hepatis, with the largest measuring approximately 2.5 cm. Multiple para‐aortic and mesenteric lymph nodes were also observed, with the largest measuring approximately 1.3 cm. Finally, a calcified lesion measuring about 2.5 × 1.3 cm was seen in segment 4a, consistent with a calcified hydatid cyst (Figure 4).

## 4 TREATMENT AND SURGICAL APPROACH

Based on these findings, the patient underwent laparoscopic surgery, during which a large, infected hydatid liver cyst occupying the right lobe and communicating with the biliary tree was identified. A suspected slipped cyst via the CBD was also noted. Approximately 200 cc of pus was aspirated from the cyst and sent for microbiological analysis. The cyst was de‐roofed, and the daughter cyst wall was removed.

## 5 FOLLOW‐UP

The patient was closely observed in the postoperative setting and experienced smooth recovery without complications. The patients were discharged after several scheduled follow‐up visits to the outpatient clinic. The subsequent liver function test results and bilirubin levels were normal, and the patient had a good prognosis.

## 6 DISCUSSION

HHC can be classified based on the mechanism of rupture or drainage of cystic fluid into the biliary duct. The former categorizes it as contained, communicating, or direct rupture, whereas the latter classifies it as occult or frank rupture depending on whether cystic fluid or intracystic material is draining into the biliary duct.^1^, ^11^ Rupture frequencies vary by location, with the right hepatic duct being the most affected (55%–60%) followed by the left hepatic duct (25%–30%).^1^

Rupture of the IBHC follow factors leading to increased intracystic pressure, degeneration of the parasitic membrane, host defense mechanisms and trauma.^12^ However, rupture inside the biliary tree duct is rare, and may lead to life threatening biliary blockage (50% mortality) and cirrhosis.^13^

Diagnosis of liver echinococcosis involves a combination of imaging, serological, and immunological tests. MRCP is particularly effective in visualizing the cystic component and supporting the diagnosis.^14^ MRCP is highly effective in identifying extrahepatic extension, vascular involvement, and biliary tree involvement and it is the procedure of choice for hepatic hydatid cyst.^15^

In our case, it revealed a distinctive beaver‐tail liver appearance, indicating cystobiliary communication between the HHC located in segments IV and V and the biliary tree; however, Avcu et al and Aghajanzadeh et al reported ruptured HHC in segment II, which is the most common liver segment to have a ruptured HHC.^16^, ^17^ The presence of daughter vesicles within biliary ducts can result in different complications like obstruction,^16^, ^17^ cholecystitis,^16^ choledochocystitis, and acute cholangitis^17^ (Table 1). An enlarged gallbladder is a common complication in such cases, either with wall thickening,^18^ or without^16^, ^17^; however, delayed time of detection might be the cause of wall thickening in some cases. Moreover, concomitant rupture of HHC in the gallbladder and intrabiliary duct is rare.^16^, ^18^

**TABLE 1:** Characteristics of previously reported cases.

	Becker et al., 1997	Avcu et al., 2009	Wani et al., 2010	Aghajanzadeh et al., 2021	Our case
Gender (Age)	Male (48)	Female (66)	Female (65)	Male (21)	Male (24)
Clinical presentation	‐Upper abdominal pain ‐Tenderness ‐Jaundice	‐Upper abdominal pain ‐Tenderness ‐Jaundice	‐Upper abdominal pain ‐Tenderness ‐Jaundice	‐Upper abdominal pain ‐Tenderness ‐Jaundice	‐Upper abdominal pain ‐No tenderness Jaundice
diagnostic tests and imaging	‐CT ‐US ‐ERCP ‐Immune hemagglutination ‐No MRCP	CT scan ‐US MRI ‐MRCP	‐No CT ‐US ‐Upper endoscopy ‐ERCP ‐ MRCP	‐CT ‐US ‐MRCP	‐CT ‐US ‐MRCP
Treatment approaches	No surgical intervention (catheter retraction) and mebendazole	Surgical intervention	Surgical intervention and albendazole.	Surgical intervention	Surgical intervention
Complications	None	None	None	Peritonitis, pancreatitis Hepatitis due to albendazole toxicity	None

CT scans are useful for observing calcifications of the cyst wall, internal septa, daughter vesicles, and identifying any disseminated cysts.^15^ We identified cyst calcifications using CT as well as in Becker's case.^14^ Conversely, Aghajanzadeh et al and Avcu et al find normal cyst wall without calcifications.^16^, ^17^

An intact cyst is naturally resistant to infection owing to the avascularity of the pericyst and limited interaction with the host blood system. However, once the cyst ruptures, bacterial infections become possible,^11^ approximately 25% of the ruptured cysts become infected.^12^ We observed a dense material within the hepatic biliary tree, indicating an intrabiliary ruptured hepatic hydatid cyst with a superinfection.

While surgery is the treatment of choice for IBHC, and it was used approach in studies,^16^, ^17^, ^18^ an alternative approach was followed in Becker's study as the patient refused surgery.^14^ Therefore, the patient underwent catheter retraction of the CBD and bile, accompanied by pus drainage from the ampulla of Vater. Oral mebendazole treatment was initiated, which resulted in steady improvement. Follow‐up after eight months revealed minor fibrosis of the liver cyst and normal antibody levels.^14^ Conservative surgery, including partial cyst resection, removal of cyst contents, and sterilization of the remaining cavity, was performed in our case and in studies.^16^, ^17^, ^18^ Wani et al gave albendazole as part of postoperative management.^18^ Our patient and patients in studies,^16^, ^17^, ^19^ were not prescribed albendazole. Albendazole is superior to mebendazole in terms of cyst degeneration, cure rates, and shorter treatment duration.^20^ The cure rates for hydatid cysts treated with albendazole alone for 3 months are lower (less than 60%) compared to surgery with postoperation albendazole treatment (>90%). However, surgery is the standard intervention for most cases, it is contraindicated in certain cases including complex or widespread injury, advanced age, pregnancy, comorbidities, multiple difficult‐to‐access cysts, and patient refusal of surgery.^20^

To the best of our knowledge, our case is the first to report an intra‐biliary hydatid cyst rupture with superinfection in the literature. Becker et al. reported the first case of an intrabiliary hydatid cyst rupture case^14^ and Wani et al. reported an atretic hepatic hydatid cyst with infected bile, which is the only case to culminate with pancreatitis.^18^

In conclusion, our case highlights the rare occurrence of intrabiliary hydatid cyst rupture with superinfection. Imaging techniques, such as CT tomography and MRCP cholangiopancreato‐graphy, play a crucial role in diagnosing and evaluating IBHC. Treatment choice depends on various factors, with surgery being the standard intervention. However, alternative approaches may be considered, based on patient preferences and specific circumstances. To the best of our knowledge, this is the fifth case, contributing to the limited literature on intrabiliary hydatid cyst rupture.

## AUTHOR CONTRIBUTIONS

**Hanan Al‐Asbahi:** Writing – original draft; writing – review and editing. **Jaber H. Jaradat:** Writing – original draft; writing – review and editing. **Mohammad Abu‐Jeyyab:** Writing – original draft; writing – review and editing. **Ruba Al‐Dwairi:** Writing – original draft; writing – review and editing. **Baraa W. Tailakh:** Writing – original draft; writing – review and editing. **Rand A. Almadadha:** Writing – original draft; writing – review and editing. **Ibraheem M. Alkhawaldeh:** Writing – original draft; writing – review and editing. **Abdulqadir J Nashwan:** Writing – original draft; writing – review and editing.

## FUNDING INFORMATION

This study received no funding.

## CONFLICT OF INTEREST STATEMENT

The authors declare that they have no conflicts of interest.

## ETHICS STATEMENT

Ethical approval was not required for this study in accordance with local or national guidelines.

## CONSENT

Written informed consent was obtained from the patient's legal guardian to publish this report, in accordance with the journal's patient consent policy.

## Supporting information


Data S1.

## Data Availability

All data generated or analyzed in this study are included in this published article. Further inquiries can be directed to the corresponding authors.
